# Indium arsenide quantum dots: an alternative to lead-based infrared emitting nanomaterials

**DOI:** 10.1039/d2cs00490a

**Published:** 2022-11-21

**Authors:** Houman Bahmani Jalali, Luca De Trizio, Liberato Manna, Francesco Di Stasio

**Affiliations:** Photonic Nanomaterials, Istituto Italiano di Tecnologia Via Morego 30 16163 Genova Italy francesco.distasio@iit.it; Nanochemistry, Istituto Italiano di Tecnologia Via Morego 30 16163 Genova Italy

## Abstract

Colloidal quantum dots (QDs) emitting in the infrared (IR) are promising building blocks for numerous photonic, optoelectronic and biomedical applications owing to their low-cost solution-processability and tunable emission. Among them, lead- and mercury-based QDs are currently the most developed materials. Yet, due to toxicity issues, the scientific community is focusing on safer alternatives. In this regard, indium arsenide (InAs) QDs are one of the best candidates as they can absorb and emit light in the whole near infrared spectral range and they are RoHS-compliant, with recent trends suggesting that there is a renewed interest in this class of materials. This review focuses on colloidal InAs QDs and aims to provide an up-to-date overview spanning from their synthesis and surface chemistry to post-synthesis modifications. We provide a comprehensive overview from initial synthetic methods to the most recent developments on the ability to control the size, size distribution, electronic properties and carrier dynamics. Then, we describe doping and alloying strategies applied to InAs QDs as well as InAs based heterostructures. Furthermore, we present the state-of-the-art applications of InAs QDs, with a particular focus on bioimaging and field effect transistors. Finally, we discuss open challenges and future perspectives.

## Abstract 2

Colloidal indium arsenide quantum dots are promising RoHS-compliant building blocks for near infrared photonic, optoelectronic and biomedical applications.

## Graphical abstract

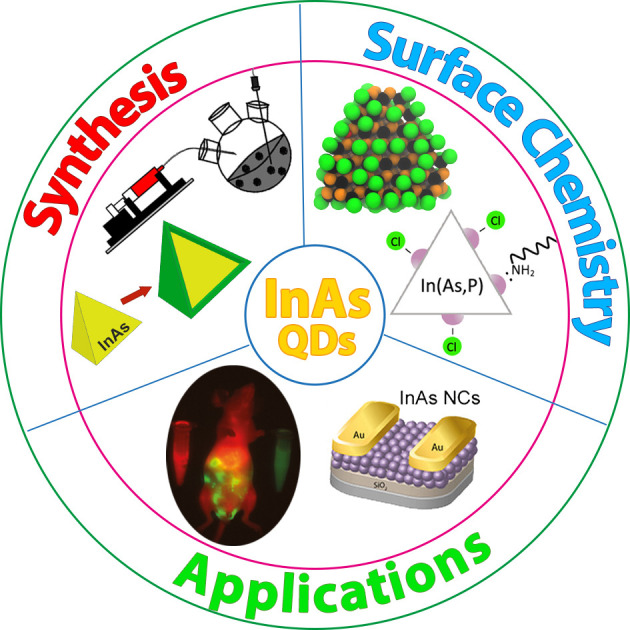

**Key learning points**

(1) History and evolution of the colloidal synthesis of indium arsenide QDs.

(2) Recent advances in chemical strategies for synthesis improvement of indium arsenide QDs.

(3) Understanding of the surface chemistry and trap passivation of indium arsenide QDs.

(4) Recent biomedical, electronic and optoelectronic applications of indium arsenide QDs.

(5) Perspectives and challenges of the colloidal indium arsenide QDs in the future.

## Introduction

Colloidal quantum dots (QDs) have already demonstrated their applicability in consumer electronic products operating in the visible spectral range.^1,2^ Yet, significant developments are still required for QDs to become applicable in the next generation of infrared (IR) technologies due to the challenges of both synthesis and optical characterization in that region of the spectrum.^3,4^ Also, there are much fewer available QDs active in the IR range compared to semiconductor QDs active in the visible range. Among them, the most studied ones are lead- and mercury-based QDs in terms of synthesis development,^5,6^ compositional modulation,^7^ band-gap engineering,^8–10^ and surface passivation.^11–14^ These “classical” IR QDs can now be easily synthesized and surface functionalized to achieve a strong and narrow photoluminescence (PL) emission. However, their use in the next-generation optoelectronic applications is severely restricted by their inherent toxicity.^15^

**Fig. 1:**
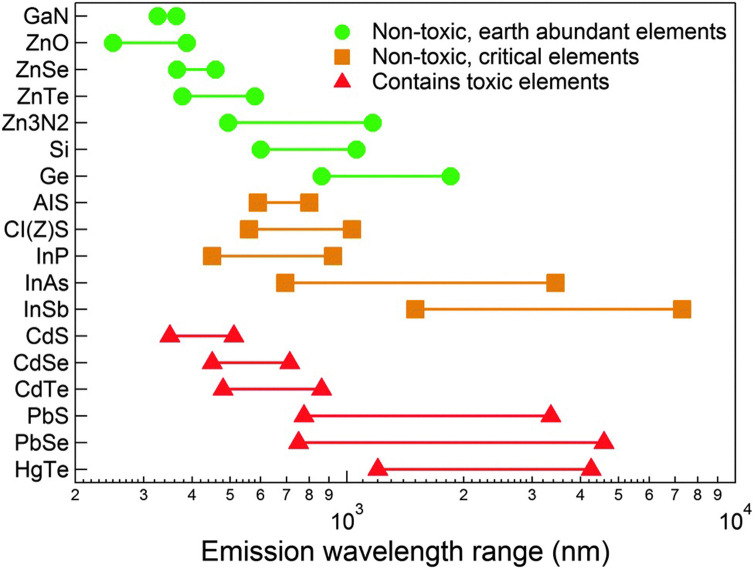
Emission wavelength range of different semiconductors in the visible and IR spectral windows. InAs is the only RoHS-compliant semiconductor that can emit in the full range of the NIR. InAs is considered critical due to the limited supply of indium element. Reprinted with permission from ref. 64, Copyright 2015, The Royal Society of Chemistry.

In this context, a promising alternative IR emitter is indium arsenide (InAs), a III–V semiconductor with a narrow bulk bandgap of 0.35 eV at room temperature (0.40 eV at 77 K),^16^ a zinc blende crystal structure (lattice constant of 6.05 Å)^16^ InAs is characterized by In–As bonds that are more covalent than those of II–VI semiconductors (*i.e.* the Philips ionicity of InAs is 0.36^17^ while those of CdSe and PbS are 0.70^17^ and 0.77,^17^ respectively). The high covalency of the In–As bond has several consequences on the physical properties of this material.^17^ First, in a covalent material such as InAs, carriers are more efficiently screened than in more ionic materials, and this translates into a large exciton Bohr radius for InAs (the size of which is still “debated” and has been reported to be ∼45 nm^18^ or ∼31 nm^19^). This means that a size dependence in the optical properties of InAs QDs is also found in relatively large crystals, a feature which provides a handle for tuning such properties across a broad size range, deep in the near infrared (NIR). Also, covalent bonds are less susceptible to be ruptured by polar molecules, such as water, compared to more ionic bonds, thus leading to an overall improved chemical stability,^17,20,21^ especially with respect to leaching of As species in aqueous environments compared for example to the more ionic Cd, Pb and Hg based semiconductors. In semiconductors, the presence of surface states is very detrimental for the optical properties due to unpassivated surface dangling bonds, as photogenerated carriers are easily trapped in them. The depth of these trap states, starting from relatively shallow levels in strongly ionic semiconductors, increases with the increasing covalency of the bond.^17^ So, it is critical for III–V QD systems to have their surface well passivated, for example by an epitaxial shell growth of a larger band gap material. It is also worth reminding that InAs is the only “Restriction of Hazardous Substances” (RoHS) compliant semiconductor^22^ having tunable optical absorption and emission in the whole near infrared (NIR) spectral range (Fig. 1). Yet, despite the comparatively lower toxicity of InAs compared to Pb- and Cd-based materials,^23^ the leaching of inherent-toxic indium^24^ and arsenic^25^ elements can still lead to health and environmental risks, so effective surface passivation approaches are needed also to tackle this issue.^26^

The optical characteristic of InAs QDs makes them promising candidates for efficient photovoltaic heterojunctions,^27^ quantum light emitting diodes^28^ and biological imaging.^29,30^ They have an absorption cross-section per dot at excitonic peak around 3.15 × 10^−16^ R^1.28^ cm^2^, estimated by Yu *et al.*,^31^ corresponding to a reduced radius-dependency than PbS QDs (7.494 × 10^−17^ R^2.31^ cm^2^).^32^ On the other hand, InAs QDs are suitable for optical communication^33^ and deep tissue bioimaging^34^ since they can emit in the range of 1300–1550 nm, where the transmission losses are minimized. Moreover, InAs QDs feature n-type conductivity in ambient atmosphere due to their electron donating surface states,^27,35–38^ unlike lead chalcogenides that switch from n-type to p-type upon air exposure.^35,39–41^ A NIR absorbing QD with n-type characteristic can be coupled to various p-type light-harvesting materials and used for efficient heterojunction solar cells.^27^

Despite the important role that InAs QDs could play in such applications, currently their in-device performance is limited due to their poor optical properties.^3,4^ The PL linewidth and PL quantum yield (PLQY) of InAs-based QDs indeed lag far behind those of the “state-of-the-art” lead sulfide (PbS)^7,9,42–45^ and lead selenide (PbSe)^45,46^ core/shell QDs.^47^ The reason is the more challenging synthesis of InAs compared to that of II–VI (CdSe,^48–51^ CdTe^49,52^ and CdS^49,53^) and IV**–**VI QDs (PbS,^54–57^ PbSe^39^ and PbTe^58^), for which a wide range of precursors, with a very tunable reactivity, is available to control both the size and size distribution, even at low temperatures.^17,21^ Moreover, the few available arsenic precursors are very reactive, making it hard to have a good control over the nucleation and growth of the nanocrystals (NCs).^21,59,60^ Consequently, broad size distributions are usually obtained, resulting in broad absorption and PL peaks. Furthermore, the temperature required to grow InAs QDs with good crystallinity (>240 °C) is higher than that of II–VI HgTe (<150 °C)^6^ and II–VI PbS (<210 °C)^61^ QDs due to the more covalent nature of the In–As bonds. To obtain efficient emitters, the surface of InAs QDs should be overcoated with a shell of an inorganic material (forming core/shell heterostructures) capable of yielding a Type-I band alignment (see “Surface chemistry and trap passivation” section for more details). Unfortunately, InAs has a large lattice parameter (6.06 Å)^20^ compared to that of many Cd-free wide bandgap materials such as ZnSe (5.66 Å),^62^ ZnS (5.42^62^ Å) and ZnO (5.20 Å).^63^ Hence, the overgrowth of a shell of these materials results in heterostructures (*e.g.* InAs/ZnS or InAs/ZnSe) that are highly strained and that typically feature a low PLQY due to the presence of interfacial defects. Therefore, any significant advancement in the synthesis of InAs QDs capable of improving these figures of merit will have a strong technological impact on various areas of IR technology.

In this tutorial review, we summarize the recent progress on colloidal InAs QDs. The review is structured in four main sections: (I) synthesis methods. These are discussed in detail, highlighting their strengths and weaknesses; (II) InAs surface chemistry, InAs based core/shell systems and their optical characteristics; (III) electronic properties and carrier dynamics of InAs QDs; (IV) applications of InAs QDs in bioimaging, field effect transistors (FETs), NIR light emitting diodes (LEDs) and photovoltaics. Finally, we provide an outlook on the future research directions in this field.

## Synthesis of colloidal InAs QDs

### Syntheses based on tris(trimethylsilyl)arsine (TMS-As) precursor, and analogues

The first wet-chemical synthesis route to InAs was reported by Wells *et al.* in 1989, and consisted of a dehalosilylation reaction (eqn (1)).^65^ Such dehalosilylation route represented a milestone for the synthesis of III–V semiconductors, as it opened up a solution phase chemical route. Indeed, prior to that, III–V compounds were synthesized *via* organometallic chemical vapor deposition (OMCVD)^66^ and molecular beam epitaxy (MBE)^67,68^ only, as no wet chemical routes had yet been developed. In that work, Wells *et al.* stirred a solution of indium chloride (InCl_3_) and tris(trimethylsilyl)arsine (TMS-As) in pentane at room temperature for 3 days, after which the solution was kept at 70–75 °C for 4 days. Such reaction releases directly As^3−^ monomers which react with In^3+^ cations (to form InAs), with the concomitant formation of Me_3_SiCl (Me stands for methyl), a low boiling compound.^65^ Finally, the solution was heated to 150 °C overnight to remove the volatile Me_3_SiCl byproduct. The final product was InAs with 98% purity. The same group later effectively eliminated the Me_3_SiCl byproduct through an annealing step at higher temperature (400 °C) and obtained 99.65% pure (albeit non-emissive) nanocrystalline InAs.^69^

1 Me_3_SiCl–As + InCl_3_ (75 °C) → InAs + 3 Me_3_SiCl

**Fig. 2:**
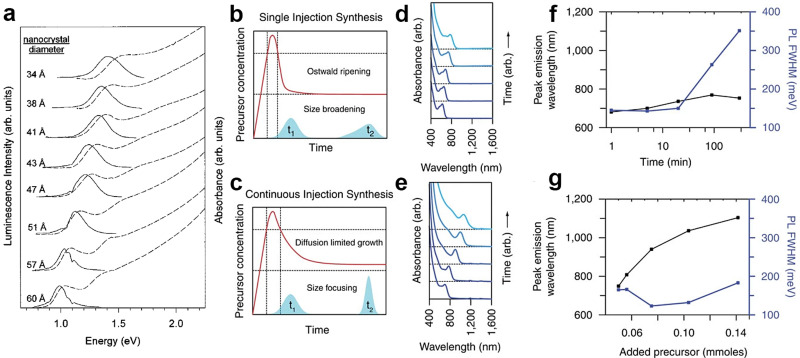
**Synthesis of colloidal InAs QDs *via* TMS-As, and analogues**. (a) The first size-tunable InAs QDs synthesized *via* the hot-injection technique using TOP as both solvent and capping agent. Reprinted with permission from ref. 70, Copyright 1996, AIP Publishing. Growth mechanism (b and c) of InAs QD synthesized *via* typical single hot-injection and continuous hot-injection techniques. Absorbance (d and e), PL tunability and PL width (f and g) of InAs QD synthesized *via* (d and f) a typical single hot-injection and (e and g) continuous hot-injection techniques. Reprinted with permission from ref. 30, Copyright 2016, Springer Nature.

Few years later, in 1996, Guzelian *et al.* used trioctylphosphine (TOP) as both solvent and capping agent and performed the same heat-up strategy at higher temperature (240–265 °C) than the 75 °C of the work of Wells *et al.*^70^ Upon size selection of the products, they could isolate InAs QDs with size-tunable band-edge emission in the 850–1200 nm range and demonstrated the presence of quantum confinement in InAs QDs for the first time (Fig. 2a). Further improvements in the synthesis of InAs QDs were made possible by employing the so-called hot-injection method: Peng *et al.* and Battaglia *et al.* in 1998 injected TMS-As into a hot solution of indium acetate [In(Ac)_3_], carboxylic acids (*i.e.* palmitic acid and myristic acid) and 1-octadecene (1-ODE) to obtain InAs QDs.^71,72^ This approach became the standard way to produce InAs QDs and it has since then been further optimized.^29,73,74^

#### Size and size distribution control of InAs QDs synthesized *via* TMS-As, and analogues

Tuning the size and simultaneously achieving a narrow size distribution is an important challenge for the colloidal synthesis of III–V QDs.^2,21,75^ The size distribution of the QDs is correlated to the width of their first exciton absorption peak, normally measured as half width at half maximum (HWHM). Since the first colloidal synthesis of InAs QDs by the Wells’ dehalosilylation reaction,^65^ the community has explored the reactions between InX_3_ (where X = halide or carboxylate) and TMS-As seeking to improve the size distribution of InAs QDs.^30,71,76,77^ The problem with TMS-As is its high reactivity (*i.e.* high conversion rate) that leads to a fast nucleation of InAs QDs, hence a fast depletion of monomers, which are then not available for the growth step. As a consequence, InAs QDs tend to grow in the Ostwald ripening regime (*i.e.* shrinking and dissolution of small particles, with the released monomers that then feed larger particles, a process that quickly leads to a broadening of the size distribution).^21,59,72,78^ Efforts to tune the precursors conversion kinetics to achieve temporal separation between the nucleation and the growth stages have only been moderately successful in the case of InAs QDs. In this regard, Harris *et al.* and Franke *et al.* investigated the effect of using TMS-As analogues, namely tris(isopropyldimethylsilyl) arsine^76^ and tris(trimethylgermyl)arsine (TMGe-As)^60^ in the synthesis of InAs QDs. Their results unexpectedly showed that changes in precursor reactivity, even up to 2–3 orders of magnitude, have negligible effects on the growth kinetics and size broadening of the InAs QDs.^76^ In another study, Franke *et al.* obtained large InAs QDs with relatively narrow size distributions (PL linewidth <135 meV) by prolonging the size-focusing regime using a multiple injection approach (Fig. 2b and c).^30^ In details, they first nucleated InAs QDs by employing a sub stoichiometric amount of As precursor (As/In ratio of 10%, using TMGe-As and indium(iii) acetate) and then grew the formed nuclei by the addition of the residual amount of arsenic precursor, using a syringe pump. This strategy led to size tunable InAs QDs with PL peak ranging from 700 nm to 1200 nm without any size-selective purification step (Fig. 2d–g).

### Syntheses based on trisdimethylamino arsine (amino-As) precursor

Since TMS-As and analogues, as well as arsine (AsH_3_),^79,80^ are highly pyrophoric, reactive, hazardous and relatively expensive,^72,81^ a few alternative arsenide precursors, including triphenylarsine (AsPh_3_),^82^ arsenic silylamide ([(Me_3_Si)_2_N]_2_AsCl)^83^ and [*t*Bu_2_AsInEt_2_]_2_^84^ have been explored. However, they all led to polydisperse InAs QDs with low PLQY. On the other hand, Green *et al.* used a safe, cheap and commercially available arsenic precursor, namely tris(dimethylamino)arsine (amino-As) for the first time.^85^ They were able to synthesize InAs QDs by thermolysis of InCl_3_ and amino-As in 4-ethylpyridine at 167 °C for up to 6 days. Griegel *et al.* improved then this technique by combining amino-As as the arsenic precursor with tris(dimethylamino)phosphine (amino-P) as a reducing agent for the synthesis of InAs QDs (the reaction was carried out at 190 °C for 30 min).^86^ The synthesis consists of two step: (i) the addition of amino-As to a mixture of indium and zinc halides dissolved in oleylamine (OAm), which generates dimethylamine and tris(oleylamino)arsine, As(NHOl)_3_, *via* a transamination reaction (eqn (2); (ii) the addition of a reducing agent, amino-P in this specific case, such that phosphorous is oxidized from +3 to +5, to trigger the nucleation of InAs QDs (eqn (3). This second step is crucial, as As(+III) species have to be reduced to As(−III) (see the below paragraph for more details). The overall synthesis scheme is the following:

2 Amino-As + 3OlNH_2_ → As(NHOl)_3_ + 3Me_2_NH

3 InCl_3_ + As(NHOl)_3_ + P^3+^ + (NHOl)_3_ → InAs + 3P^5+^ + (NHOl)_4_ Cl

#### Role of reducing agents in the synthesis of InAs with amino-As precursor

**Fig. 3:**
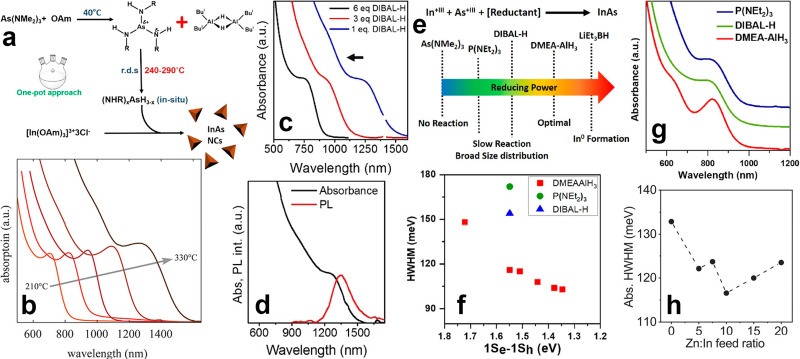
**Synthesis of colloidal InAs QDs *via* amino-As**. (a) The mechanism regulating the formation of InAs QDs using DBAL-H as reducing agent. Reprinted with permission from ref. 89, Copyright 2016, American Chemical Society. (b) Excitonic absorption peaks *versus* reaction temperature of the colloidal InAs QDs synthesized *via* indium(i) chloride as both indium source and reducing agent. Reprinted with permission from ref. 99, Copyright 2020, American Chemical Society. (c) Optical absorption spectra of InAs QDs grown at 240 °C for 5 min with different concentrations of DIBAL-H. (d) Optical absorption and PL spectrum of ∼5 nm InAs QDs. (e) Schematic representation of the reducing power of different reducing agents used for the synthesis of InAs QDs *via* amino-As. Reprinted with permission from ref. 89, Copyright 2016, American Chemical Society. (f) HWHM and (g) absorption spectra of InAs QDs synthesized using different reducing agents. Reprinted with permission from ref. 90, Copyright 2018, American Chemical Society. (h) HWHM of InAs QDs synthesized by different Zn : In feed ratios. Reprinted with permission from ref. 100, Copyright 2022, American Chemical Society.

The addition of the reducing agent is of paramount importance for the synthesis of InAs QDs with amino-As, since this compound is not able to undergo disproportionation, unlike what observed for amino-P [*i.e.* 4 P(+III) → P(−III) + 3 P(+V)].^86–89^ In the synthesis of InAs QDs by amino-As, the reducing agent governs the release of As^3−^ monomers and therefore the nucleation and growth kinetics. In this regard, Srivastava *et al.* replaced amino-P with diisobutylaluminum hydride (DIBAL-H) (Fig. 3a) and synthesized size tunable InAs QDs with a broad excitonic absorption peak in the range of 750–1450 nm (Fig. 3c and d).^89^ To optimize the synthesis kinetics, the same group tested other types of reducing agents, namely amino-P, Alane *N*,*N* dimethylethylamine complex (DMEA-AlH_3_), DIBAL-H and lithium triethylborohydride (LiEt_3_BH), and monitored the corresponding InAs products.^90^ According to their study, the precursors conversion rate could be controlled by tuning the reducing strength of the reducing agent and so the size and size distribution of the InAs QDs (Fig. 3e–g).^90^ In details, LiEt_3_BH (the strongest reducing agent tested) led to the formation of undesired metallic indium particles together with large InAs crystallites.^90–92^ Conversely, amino-P (the weakest reducing agent tested) delivered InAs QDs with a broad size distribution (HWHM of 170 meV), as evidenced by the almost featureless absorption peak of the resulting product.^90^ On the other hand, DMEA-AlH_3_, which features a slightly higher reducing power than DIBAL-H, was found to ensure the best control over the size distribution of InAs QDs, with HWHM as low as 120 meV^90^ (Fig. 3g). Furthermore, it was observed that amino-P could be activated only at elevated temperatures (240–280 °C), while DIBAL-H or DMEA-AlH_3_ can form amorphous molecular intermediates (also called InAs clusters) even at room temperature.^90^ The list of all precursors used for the synthesis of InAs QDs *via* hot-injection is summarized in Table 1.

**Table 1:** List of chemicals used for the synthesis of InAs QDs *via* the hot-injection method. In(Ac)_3_: indium(iii) acetate, InCl_3_: indium(iii) chloride, InCl: indium(i) chloride, TMS-As: tris(trimethylsilyl)arsine, amino-As: trisdimethylamino arsine, TMGe-As: tris(trimethylgermyl)arsine, AsCl_3_: antimony chloride, As(OLA)_3_: trioleylarsane, DIBAL-H: diisobutylaluminum hydride, DMEA-AlH_3_: Alane *N,N* dimethylethylamine complex, LiEt_3_BH: lithium triethylborohydride, MA: myristic acid, PA: palmitic acid, OA: oleic acid, TOP: tri-*n*-octyl phosphine, DDPA: dodecylphosphonic acid, OAm: oleylamine, 1-ODE: 1-octadecene (pyrophoric items are marked by *)

Indium source	Arsenide source	Reducing agent	Ligand	Solvent	Ref.
In(Ac)_3_	As(TMS)_3_*	—	PA, MA, TOP	1-ODE	71 and 76
In(Ac)_3_	As(TMS)_3_*	—	TOP, LA	1-ODE	93
In(Ac)_3_	As(TMS)_3_*	—	OA	1-ODE	94 and 95
In(Ac)_3_	As (iPrDMSi)_3_*	—	MA, TOP	1-ODE	76
In(Ac)_3_	As(TMGe)_3_*	—	OA, TOP	1-ODE	30 and 76
In(Ac)_3_	As(TEGe)_3_*	—	MA, TOP	1-ODE	76
In(Ac)_3_	AsCl_3_	LiEt_3_BH*	OAm	OAm	96
InCl_3_	Amino-As	Amino-P	OAm	OAm	86 and 97
InCl_3_	Amino-As	DIBAL-H*	OAm	OAm	89
InCl_3_	Amino-As	DMEA-AlH_3_*	OAm	1-ODE	90
InCl_3_	Amino-As	LiEt_3_BH*	OAm	1-ODE	90
InCl_3_	As(OLA)_3_	DIBAL-H*	OAm	OAm	98
InCl	Amino-As	InCl	TOP, DDPA	OAm	99
In(Ac)_3_	Amino-As	DMEA-AlH_3_*	OAm + ZnCl_2_	OAm	100
InCl_3_	Amino-As	Amino-P	OAm	OAm	97 and 101

Since most of the reducing agents employed for the synthesis of InAs QDs are hazardous and/or pyrophoric (*e.g.* DMEA-AlH_3_, LiEt_3_BH and DIBAL-H), recently Ginterseder *et al.* explored the use of indium(i) chloride as both the indium source and reducing agent.^99^ This chemical has a limited toxicity and has been already used in synthetic organic chemistry as an alternative to highly reactive hydride sources.^102–104^ The authors hypothesized that the oxidation of In^1+^ to In^3+^ provides electrons for the reduction of As^3+^ to As^3−^ and the consequent formation of In–As species. By this method, it is possible to synthesize gram scale products and tune the absorption peak of the InAs QDs in the 700–1400 nm range (by varying the reaction temperature) (Fig. 3b).

### Seeded growth approach for the synthesis of larger InAs QDs

In the TMS-As based strategy, the control over the size distribution of InAs QDs can be achieved only on small QDs, with emission wavelengths shorter than ∼900 nm, since TMS-As triggers a fast nucleation of InAs QDs. In this synthesis scheme, an almost complete depletion of monomers takes place at the early stages of the synthesis, thus preventing the further QDs growth in the size focusing regime. In the amino-As route, it is possible to modulate the reactivity of the As precursor by carefully adjusting parameters such as the reducing power of the reducing agent and the temperature. With the aim of synthesizing large QDs, Leeman *et al.* lowered the injection temperature of amino-As to 170 °C (compared to 270 °C previously employed by the same group^97^) and increased the volume of the solvent (*i.e.* to reduce the concentration of monomers in order to achieve a larger nucleation radius)^105^ eventually obtaining larger nuclei and consequently larger InAs QDs.^101^ The resulting InAs QDs exhibited a band-edge absorption tunable from 1140 nm to 1400 nm with relatively broad HWHM (∼150 meV).

**Fig. 4:**
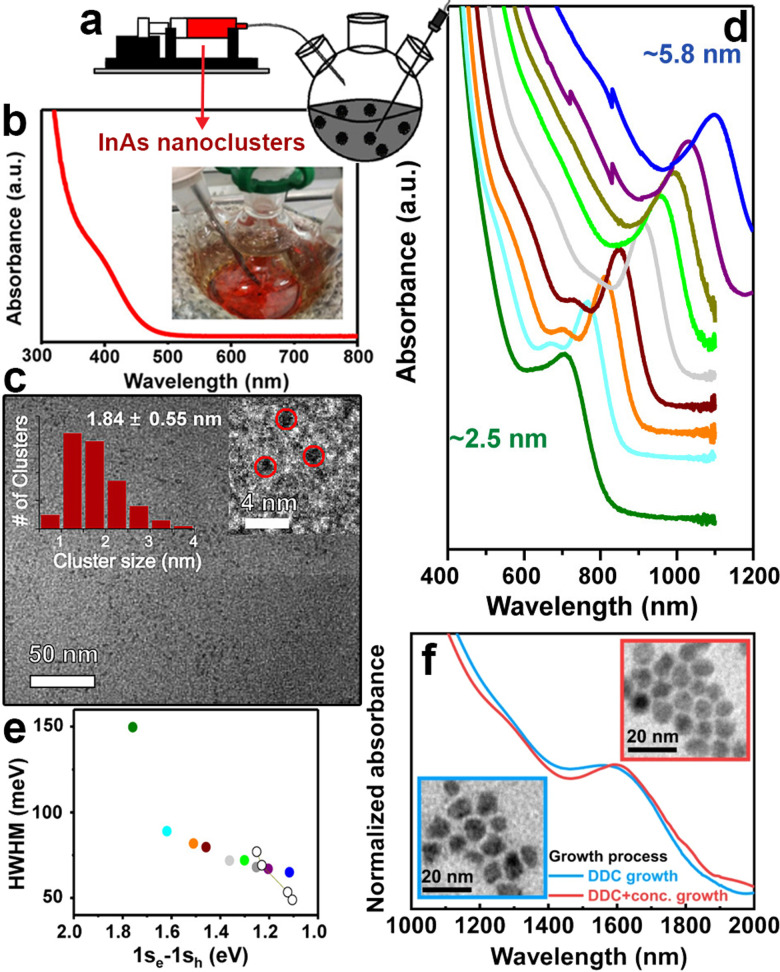
**Synthesis of large InAs QDs *via* the seeded growth method**. (a) Slow and continuous injection of amorphous InAs nanoclusters into InAs QD seeds using a syringe pump. (b) Absorption spectra and (c) TEM image of small amorphous InAs nanoclusters. (d) Absorption spectra of differently sized InAs QDs synthesized *via* the controlled injection of amorphous nanocluster solution (at 0.005 mmol min^−1^, temperature 270 °C) (e) HWHM of the first excitonic peak of InAs QDs synthesized by adding 0.05 to 3 mmol (filled circles) of precursor solutions compared with the initial seed solution (green). Empty circles represented literature values for high-quality PbS QDs for comparison. Reprinted with permission from ref. 108, Copyright 2016, American Chemical Society (f) Absorption spectra of InAs QDs grown using diffusion-dynamics-control (DDC) for 9 h, with excitonic peaks at 1579 (blue line) and optimized DDC with increased precursor concentration for 10h, with excitonic peak at 1600 nm (red line), respectively (inset: TEM images of these InAs QDs). Reprinted with permission from ref. 109, Copyright 2021, Springer Nature.

While the one-pot synthesis approach to produce large InAs QDs with narrow size distribution still requires further developments, other approaches have shown interesting results. In this regard, the so-called “seeded-growth” strategy, performed either with TMS-As or with amino-As precursors, can be used to grow large InAs QDs with good control over the size distribution. In this method, precursors are added to a dispersion of pre-formed QDs (also called seeds) to promote their growth while avoiding the nucleation of new crystals, so that the growth rate of the seeds can be controlled by tuning the addition rate of the precursors.^2,106,107^ These precursors are clusters bringing the advantage of containing both In^3+^ and As^3−^ species that are readily available to react with the growing seeds. These clusters can be produced with either TMS-As (reacting the latter with indium oleate at room temperature,^108,109^Fig. 4b) or amino-As (*via* its reaction at room temperature with both InCl_3_ and reducing agents such as DIBAL-H or DMEA-AlH_3_). For example, Tamang *et al.* synthesized 2.5 nm sized InAs seeds (Fig. 4c) *via* TMS-As that exhibited an excitonic peak at 1.77 eV and HWHM larger than 100 meV (Fig. 4b). Then, they grew these seeds *via* the addition of InAs clusters at various temperatures (270–300 °C) and at different injection rates (using a syringe pump, Fig. 4a) and obtained InAs QDs with a rather narrow size distribution (HWHM <80 meV, Fig. 4e), in the size range of ∼3 to 6 nm (Fig. 4d). In another study, Kim *et al.* optimized the QDs growth in the size focusing regime and synthesized 9 nm sized InAs QDs with an even narrower size distribution (HWHM of 60.5 meV) having excitonic peak at 1600 nm (Fig. 4f) by carefully engineering the injection rate of InAs clusters.^109^

### Shape control of InAs QDs

Tuning the shape of QDs represents an effective way to tailor their chemical, physical, and optical properties.^2^ A practical way to manipulate the shape of colloidal QDs relies on the use of specific surfactants. In details, the several facets that a QD can expose are characterized by different surface energies and chemical behaviours (*i.e.* different types and geometric arrangements of atoms and of broken bonds), often related to anisotropies in the crystal structure.^105,110^ The higher affinity of a specific surfactant to some facets than others can affect the corresponding growth rates, leading to QDs featuring non-symmetric shapes, as extensively reported for CdSe^111–113^ and InP^114^ QDs. In the case of InAs QDs, Trentler *et al.* synthesized nanorods(NRs)/nanowires(NWs) for the first time by employing thiophenol.^92^ In other works, metal nanoparticles of either indium,^115^ gold,^110,115^ bismuth^116–118^ or silver^115^ have been used to promote the nucleation and growth of anisotropic InAs nanostructures. For example, Kan *et al.* synthesized InAs NRs by injecting dodecanethiol-stabilized gold nanoparticles with diameter of ∼2 nm into a solution of InCl_3_, TMS-As and TOPO at high temperature (360 °C).^110^ The resulting InAs NRs had a diameter of 4.1 nm and variable lengths (ranging from 9.4 nm to 22.4 nm) the latter varying as a function of the added gold concentration. The InAs NRs exhibited length-dependent optical properties and a reduction in the PL intensity and a red-shift of the bandgap (up to 120 meV) were seen by increasing the rod length.^110^ In InAs NRs, the length is governed by a strongly quantum-confined regime, unlike CdSe NRs in which a medium to weak confinement regime is observed, possibly due to the larger bulk exciton Bohr radius of InAs (35 nm) compared to that of CdSe (5 nm).^110^

### Synthesis of InAs-based alloyed/doped QDs

**Fig. 5:**
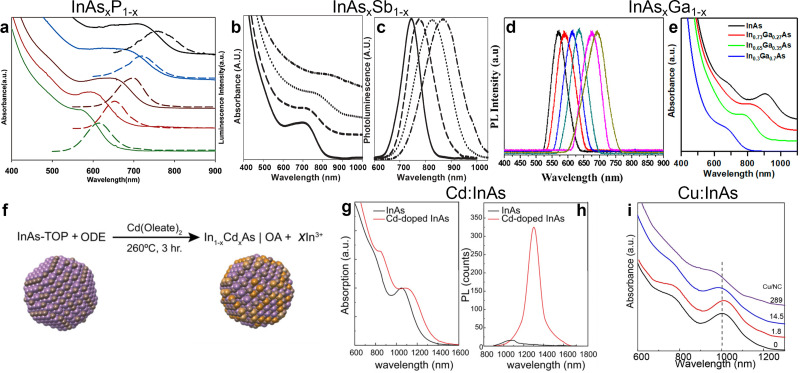
**Synthesis of alloyed/doped InAs QDs.** (a) Absorbance (solid line) and PL (dashed line) of InP (green), InAs_0.33_P_0.66_ (red), InAs_0.66_P_0.33_ (brown), InAs_0.82_P_0.18_ (blue), and InAs (black), from bottom to top, respectively. Reprinted with permission from ref. 124, Copyright 2005, American Chemical Society. (b) Absorbance and (c) PL of InAs (solid line), InAs_0.97_Sb_0.03_ (dashed line), InAs_0.90_Sb_0.10_ (dotted line), InAs_0.86_Sb_0.14_ (dash-dotted line). Reprinted with permission from ref. 123, Copyright 2006, The Royal Society of Chemistry. (d) Normalized PL of various In_1−*x*_Ga_*x*_As/ZnSe QDs; (right to left: in content 5%−30%). Reprinted with permission from ref. 122, Copyright 2016, American Chemical Society. (e) Absorption spectra of InAs (black), In_0.73_AsGa_0.27_ (red), In_0.65_AsGa_0.35_ (green) and In_0.3_AsGa_0.7_ (blue) QDs. Reprinted with permission from ref. 20, Copyright 2018, American Chemical Society. (f) Scheme of Cd doping of InAs synthesized *via* TMS-As. (g) Absorption and (h) PL of undoped (black lines) and Cd-doped (red lines) InAs QDs. Reprinted with permission from ref. 36, Copyright 2020, John Wiley and Sons. (i) Absorption spectra of undoped and Cu doped InAs QDs. Reprinted with permission from ref. 129, Copyright 2019, American Chemical Society.

Features of QDs such as band gap and PLQY are strongly dependent on the QDs’ size. However, they are also dependent on the chemical composition, and as such they can be readily tuned by synthesizing alloyed QDs.^2,119–121^ In the specific case of InAs, the incorporation of 3+ cations (Ga),^122^ or 3− anions (Sb^123^ or P^86,97,124^) leads to alloyed InAs QDs with compositionally tunable optical features. For example, Kim *et al.* synthesized InAs_*x*_P_1−*x*_ alloyed QDs (by using TMS-As and TMS-P) that had a radial graded composition, with increasing arsenic content from the center to the edge of the dots. They were able to tune the emission of the QDs from 600 nm to 800 nm by changing the As-to-P ratio while keeping the size almost unchanged (Fig. 5a).^124^ In another study, Kim *et al.* synthesized InAs_*x*_Sb_1−*x*_ QDs by the hot injection of TMS-As and TMS-Sb and observed a red-shift of the excitonic feature from ∼700 nm to ∼850 nm by increasing the Sb content, since bulk InSb has narrower band gap than InAs (Fig. 5b).^123^ However, the PL width increased from 84 nm to 164 nm when going from InAs to InAs_0.86_Sb_0.14_, indicating a poor control over the size distribution while employing the two pnictide sources together (Fig. 5c). For the case of In_1−*x*_Ga_*x*_As alloyed QD, Park *et al.* synthesized InAs_*x*_Ga_1−*x*_ by the hot-injection of TMS-As and tuned the emission of InAs from 700 nm to 580 nm (Fig. 5d), with the In_0.2_Ga_0.8_As/ZnSe featuring a PLQY of 25.6%.^122^

Another toolkit for preparing alloyed QDs is represented by cation exchange reactions, in which cations of pre-formed colloidal NCs are partially substituted with new cations, while retaining the size and shape of the QDs.^125^ This strategy has been largely employed in various II–VI, I–VI, IV−VI and even III–V systems to get either alloyed QDs or even heterostructures, including core/shell systems.^2,126,127^ In the case of InAs QDs, cation exchange reactions have been successfully exploited by Srivastava *et al.* who exchanged In^3+^ with Ga^3+^ cations in preformed InAs QDs, thus forming ternary In–Ga–As QDs with tunable bandgap.^20^ In details, the absorption of InAs_*x*_Ga_1−*x*_ QDs could be tuned from ∼950 nm to ∼750 nm by increasing the Ga content (Fig. 5e).^20^ Interestingly, the cation exchange reaction was performed in molten salts (namely, a mixture of CsBr:LiBr:KBr), a reaction environment that allows to work at relatively high temperatures (380–500 °C), at which traditional organic solvents and surface ligands would either evaporate or decompose.^128^

In terms of doping, Asor *et al.* doped the surface of InAs with cadmium by the dropwise addition of Cd(oleate)_2_ to a preheated solution of InAs QDs in 1-ODE and OAm at 260 °C under inert atmosphere (Fig. 5f).^36^ Their results indicated that cadmium doping not only increases the chemical stability of InAs QDs against oxidation under ambient atmosphere, but also transforms them from n-type to p-type (see “Applications” section for more details). As regarding the optical properties, cadmium doping led to the broadening and red-shifting (by 95 meV) of the excitonic absorption peak (Fig. 5g), as well as a red-shifting (by 190 meV) the PL peak and increase the PL intensity by an order of magnitude (Fig. 5h). In another study, Tripathi *et al.* doped InAs QDs with copper *via* the reaction of copper chloride (CuCl_2_) at room temperature.^129^ The copper doping led to an improvement in the carrier mobility (see “Applications” section for more details)^129^ and a redshift of the absorption peak (however less marked than in the case of cadmium, see Fig. 5i). This type of diffusion-based doping allows direct comparison of the electronic properties upon introducing different dopants (*e.g.* Cu,^130^ Au^131^ and Ag^37^) to InAs QDs.

## Surface chemistry and trap passivation of InAs QDs

**Fig. 6:**
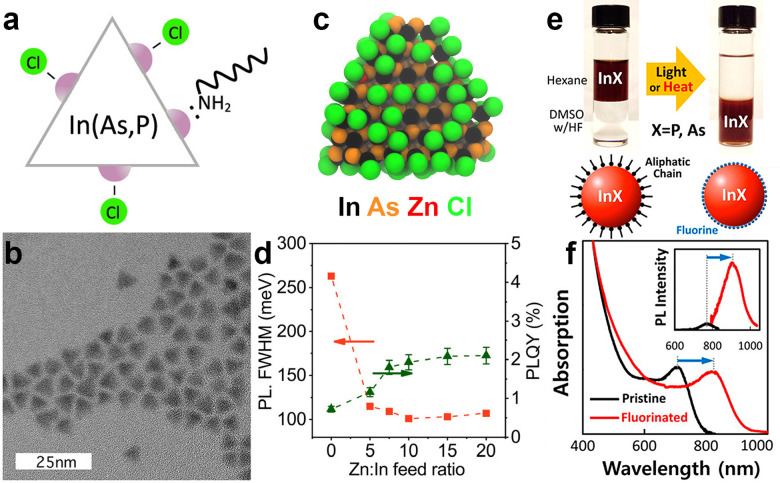
**Surface chemistry and trap passivation of InAs QDs.** (a) Schematic of the proposed surface termination and (b) TEM image of the tetradedron-shaped InAs QDs synthesized *via* Amino-As. Reprinted with permission from ref. 97 Copyright 2021, American Chemical Society. (c) Model of InAs QD synthesized *via* amino-As and passivated with ZnCl_2_. (d) The effect of Zn : In ratio on the PL fwhm and PLQY of the InAs QDs. Reprinted with permission from ref. 100 Copyright 2022, American Chemical Society. (e) Trap passivation of InAs QDs *via* surface fluorination. (f) Absorption spectra of not-passivated (black) and passivated InAs QDs *via* HF (red). (Inset: Normalized PL spectra). Reprinted with permission from ref. 18 Copyright 2018, American Chemical Society.

The large surface-to-volume ratio of QDs typically plays a detrimental role in the recombination of photoexcited carriers since surface uncoordinated atoms act as non-radiative recombination sites (*i.e.* traps).^132^ Both surface states and surface ligands play a crucial role in determining the final electronic and optical properties of InAs QDs.^100^ While the surface chemistry of “classical” II–VI and IV–VI QD systems has been studied extensively, that of InAs QDs is still under investigation. As the very first study in this direction, Leemans *et al.* examined the surface chemistry of InAs QDs synthesized with amino-As (and amino-P as the reducing agent).^97^ The InAs QDs analyzed in their work had a truncated tetrahedron shape (Fig. 6b) terminated by In(111)-facets in which In^3+^ atoms are passivated by Cl^−^ ions (X-type ligand) and OAm (L-type ligands) (Fig. 6a).^97^ In another study, the same group replaced the native, long-chain OAm ligands by a combination of short-chain stabilizers such as *n*-butylamine and 3-mercapto-1,2-propanediol to enhance the inter-dots charge carrier mobility.^101^ Recently, Zhu *et al.* revealed that most of the InAs trap states originate from surface indium vacancies. These can be partially passivated by zinc chloride (ZnCl_2_) acting as Z-type ligands (Fig. 6c) and leading to a slight improvement in the PLQY from below 1% to 2% (Fig. 6d).^100^ In another study, Kim *et al.* effectively passivated indium vacancies *via* fluoride ions using hydrofluoric acid (HF) (Fig. 6e).^18^ Surface fluorination of InAs cores red-shifts the PL (250 meV) and boosts the PLQY from 1.5% to 11% (Fig. 6f). They justified this significant PL red-shift by the InAs large exciton Bohr radius, which enables excitons to spread over a large volume, thus indicating that the optical behavior of InAs QDs is strongly susceptible to changes in the electronic structure of the surface. In addition, the electron effective mass of bulk InAs (0.024 m_0_^133^) is significantly smaller than that of other classical semiconductors such as CdSe (0.11 m_0_^133^) and InP (0.08 m_0_^134^). This entails a decreased confinement of the electron wavefunction in the InAs core region and a decrease in radiative recombination.^133^

### InAs based heterostructures and their optical characteristics

**Fig. 7:**
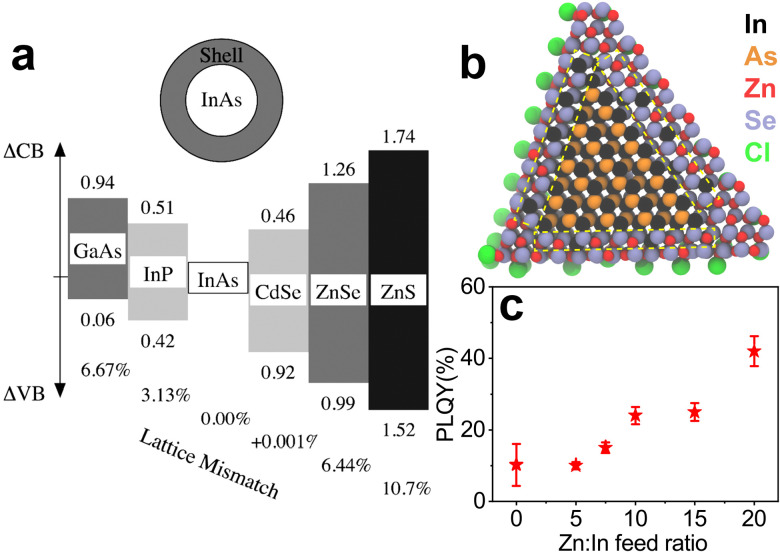
(a) Lattice mismatch and band offsets of different shelling materials suggested for InAs QDs. Reprinted with permission from ref. 135 Copyright 2000, American Chemical Society. (b) Model of a InAs/ZnSe core/shell QD with a hybrid In–Zn–Se layer between the pure InAs core and pure ZnSe outer shell. (c) PLQY of InAs/ZnSe core/shell QDs obtained from InAs cores made with different Zn : In feed ratios. Reprinted with permission from ref. 100 Copyright 2022, American Chemical Society.

The choice of shell materials for InAs is limited due to the large lattice parameters of InAs, as previously mentioned.^100^ CdSe is the most popular and effective shelling material for InAs since it has a very low lattice mismatch with InAs (0.001%)^135^ compared to ZnSe (6.44%^135^) and ZnS (10.7%^135^). Apart from the optimal lattice matching, CdSe should form Type-I band alignment with InAs considering the bulk bandgaps of the two materials (Fig. 7a). However, CdSe forms a quasi Type-II alignment with InAs, thus delocalizing the electron wavefunction in the shell region.^133,135,136^ For this reason, it is necessary to cover InAs/CdSe QDs with another shell material (*e.g.* CdS, ZnSe or ZnS) to effectively confine the photoexcited carriers. Franke *et al.* synthesized InAs/CdSe/CdS QDs with emission peaked at 970 nm and a PLQY of 82%.^30^ The same authors observed that the PLQY values of InAs/CdSe/CdS QDs decreased when increasing the size of the InAs core, with 1425 nm emissive QDs having a PLQY of 16%. Analogously, Cao *et al.* reported a PLQY of 18% and broad PL fwhm (0.2 eV) for large InAs QDs overcoated with 1.2 nm CdSe shell emitting at 1120 nm.^133^ Upon subsequent shelling of these systems with a ZnSe outer layer (forming InAs/CdSe/ZnSe heterostructures), the PLQY could be further increased to 52% (PL peak ∼ 1065 nm).^137^

InAs is considered as an alternative to toxic lead and cadmium chalcogenides, so shell materials based on Cd are not suitable to obtain RoHS-compliant emitters. In this regard, ideal shelling materials are ZnSe and ZnS, which are supposed to make a Type-I alignment with InAs (Fig. 7a). On the other hand, Zhang *et al.* showed that InAs/ZnSe core/shell structures are sensitive to air and they ascribed it to the extension of the electron wave function to the ZnSe shell.^138^ In any case, since ZnS and ZnSe have high lattice mismatch with InAs, the reported InAs/ZnS(Se) core/shell structures exhibit low PLQY values in the order of 10%.^73,86^ For these reasons, various attempts have been made to form graded intermediate shells of lattice-matching materials, such as InP, which has low lattice mismatch with InAs (3.13%).^135^ Xie *et al.* have grown InAs/InP/ZnSe QDs with a PLQY as high as 76% and PL peaked at 980 nm.^74^ Sagar *et al.* synthesized Cd-free InAs/In(Zn)P/GaP/ZnSe emitting at ∼1107 nm with a PLQY of 23%.^47^ They developed a single precursor complex to form InZnP as intermediate shell that was able to preserve the excellent size dispersion (6%) of the InAs core. In another study, InAs/In(Zn)P/ZnSe/ZnS heterostructures were prepared by the injection of In(Zn) and TMS-P precursor solutions into an InAs core solution. The QDs had a PLQY of 25% and an emission peak of 873 nm.^139^ Recently, Enright *et al.* synthesized InAs/InP/ZnSe heterostructures emitting at ∼830 nm with a PLQY >50% and found that the electron wavefunction extends isotropically into the InP shell but remains close to the InAs/InP interface, in analogy with the report of Sagar *et al.*^140^ In a recent work, our group prepared InAs/ZnSe QDs in which the lattice mismatch between the core and the shell was minimized *via* the formation of an In–Zn–Se interlayer, boosting the PLQY up to 42% with an emission at 860 nm (Fig. 7c).^100^ As mentioned earlier, Zhu *et al.* used ZnCl_2_ as additive in the InAs core synthesis, which formed an In–Zn–Se interlayer and released the strain between InAs and ZnSe (Fig. 7b) (Table 2).

**Table 2:** InAs QD based heterostructures and their optical characteristics

Core material	Core size (nm)	Shell material	Shell thickness	Excitonic peak (nm)	PL peak after shelling (nm)	fwhm (nm, meV)	PLQY (%)	Ref.
InAs	1.7	InP	n/a	886–991	n/a	n/a	n/a	135
InAs	n/a	InP	n/a	990–1082	n/a	n/a	n/a	133
InAs	n/a	InP	1–3 nm	880	905	60–75, 48–60	1.2	74
InPAs	2–4.23	InP/ZnSe	0.4 nm	580–720	625–780	68, 54	3.5	141
InAs	n/a	InP/ZnSe	2–3 nm	910	985	60–75, 48–60	76	74
InAs	2.7 ± 0.2	InP/ZnSe	1.5 nm + 2 ML	∼800	∼870	∼75, 130	60	140
InAs	n/a	InP/ZnSe/ZnS	n/a	n/a	∼775	∼150, 260	36	138
InAs	3.1	InZnP/GaP/ZnSe	n/a	∼953	∼1107	∼124, 100	23	47
InAs	3	In(Zn)P/ZnSe/ZnS	∼6.6	∼780	∼890	∼90, 72	25	139
InAs	3.4	CdSe	1.2 nm	∼1030	∼1120	∼248, 200	18	133
InAs	1.4	ZnCdS	2.9 nm	755	795	82, 66	35–50	29
InAs	3.1–5.5	CdSe	1 ML	∼800	∼850	70, 56	∼35	142
InAs	3.1–5.5	CdSe	2 ML	∼900	∼950	70, 56	∼92	142
InAs	3.1–5.5	CdSe	3 ML	∼990	∼1050	70, 56	∼85	142
InAs	3.1–5.5	CdSe	4 ML	∼1010	∼1100	70, 56	∼67	142
InAs	3.1–5.5	CdSe	5 ML	∼1025	∼1080	70, 56	∼56	142
InAs	n/a	CdSe	0.5 m	700–1400	1000–1500	∼148, 119	∼13	99
InAs	3.1	CdSe	1.8 nm	n/a	∼1320	n/a	20	143
InAs	n/a	CdSe/CdS	n/a	n/a	970	n/a	82	30
InAs	n/a	CdSe/CdS	n/a	n/a	1100	n/a	37	30
InAs	n/a	CdSe/CdS	n/a	n/a	1300	n/a	26	30
InAs	n/a	CdSe/CdS	n/a	n/a	1450	n/a	16	30
InAs	3.1	CdSe/CdS	1.8 nm	n/a	∼1362	n/a	24	143
InAs	1.9	CdSe/ZnSe	2.8	n/a	885	∼185, 149	70	137
InAs	3.8	CdSe/ZnSe	1.8	n/a	1065	∼160, 129	52	137
InAs	6.3	CdSe/ZnSe	1.5	n/a	1425	∼200, 161	2.5	137
InAs	3.1	CdSe_*x*_S_1−*x*_/CdS	4.5 nm	n/a	∼1320	∼260, 209	30	143
InAs	2–2.4	ZnSe	1.5 nm	1160–1240	1300	110–180, 88–145	n/a	144
InAs	n/a	ZnSe	n/a	645–771	750–920	85–121, 68–97	1–2	73
InAs	3.4	ZnSe	n/a	∼900	1010	147, 118	5–10	86
InAs	3.4	ZnS	n/a	∼835	925	111, 89	5–10	86
In(Zn)As	n/a	ZnSe/ZnS	n/a	490	538	60, 48	60	93
In(Zn)As	n/a	In(Zn)P/GaP/ZnS	n/a	∼775	∼850	110, 192	75	94 and 95
InGa_0.2_As_0.8_	n/a	ZnSe	n/a	500–600	580–700	∼50, 40	25.6	122
InGa_0.5_As_0.5_	n/a	CdS	n/a	∼750	825	∼100, 80	9.8	20
Zn–InAs	2.8	ZnSe	∼2 ML	∼810	860	195	42	100

## Electronic properties and carrier dynamics

Steiner *et al.* have pioneered the study of electronic properties of colloidal InAs QDs using scanning tunneling spectroscopy (STS).^145^ In a seminal paper in 1999, they were able to identify atomic-like electronic states with s and p character in InAs QDs.^146^ In the tunnelling current–voltage experiments, these states are seen as multiplets (two- and six-fold for s and p states, respectively), as each peak corresponds to the injection of a single electron in the QDs. Therefore, the injection of a second electron in a partially filled s type QD “orbital” will require an additional charging energy contribution. The same group also observed that the conduction band ground state was more red-shifted than the valence band ground state in two dimensional InAs QDs arrays, a behavior that could be easily traced to the remarkably smaller effective mass of the electrons (*m*_e_) compared to that of the hole (*m*_h_, *m*_h_/*m*_e_ = 17).^145,147^ Hence, electrons are more easily delocalized than holes across multiple QDs, leading to stronger coupling of the electron states compared to the hole states. In addition, InAs presents electron-donating surface states.^38^ All these aspects contribute to make InAs QDs one of the few n-type colloidal semiconductors in the NIR, independently of its processing, thus enabling the fabrication of photovoltaics based on rectifying junctions.^27^ Furthermore, InAs in the nanoscale held major promise for low-threshold carrier multiplication (CM) thanks to its energy band gap tunable in the NIR combined with the considerable difference in *m*_e_ and *m*_h_ (with respect to CdSe, PbS and PbSe QDs).^147,148^

The expected low-threshold CM motivated studies on core-only and core/shell InAs QDs carrier dynamics to elucidate the various multi-exciton processes (multi-exciton dynamics plays a central role in determining the applicability of QDs in a variety of optoelectronic applications). Yet, the literature on this topic is quite limited, and this can be ascribed to the typically poor optical properties of InAs QDs resulting from not yet optimized colloidal synthesis procedures.^148–152^ Transient absorption (TA) measurements have been carried out on both core-only and core/shell InAs QDs to determine the carrier cooling rate, single- and bi- exciton lifetimes combined with their relative amplitude and Auger recombination. Schaller *et al.* performed one of the first TA studies in 2007 on InAs and InAs/CdSe core/shell QDs probing the 1S transition.^148^ The authors observed pump-intensity-dependent TA dynamics from which they extracted a single-exciton lifetime of 192 ps and 205 ns and a bi-exciton lifetime of 8.3 ps and 16 ps for InAs core-only and InAs/CdSe core/shell QDs, respectively (Fig. 9a and b). In addition, they measured a carrier cooling rate of 0.5 eV ps^−1^. Sub-picosecond carrier cooling has been reported for InAs cores of different sizes as well as in core/shell QDs,^149,150,152^ and a fast carrier cooling is commonly observed in other QDs such as CdSe^153^ ones. On the other hand, bi-exciton and single-exciton lifetimes varies greatly in all reports with the former ranging from 8 to 53 ps.^148,150,151,154^

### Auger recombination

**Fig. 8:**
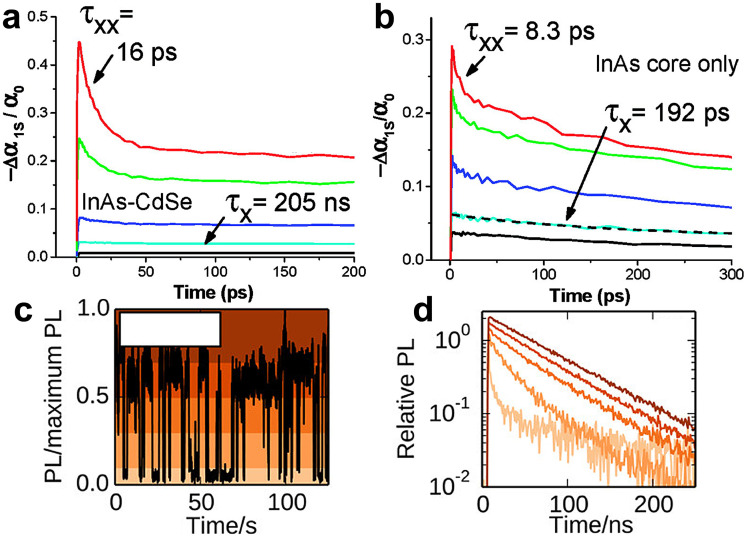
**Carrier dynamics of InAs QDs**. (a) Pump dependent TA dynamics measured for an InAs/CdSe core/shell QDs colloidal suspension (*λ*_pump_ = 800 nm, *λ*_probe_ = 1000 nm) showing a slow single-exciton relaxation at low pump intensity (black and cyan lines) and a fast relaxation component for high pump intensity (blue, green, and red lines). (b) Pump dependent TA dynamics measured for an InAs QDs suspension (*λ*_probe_ = 800 nm, *λ*_probe_ = 950 nm) showing a constant single-exciton dynamic with constant decay. Reprinted with permission from ref. 148 Copyright 2007, American Chemical Society. (c) PL of a single InAs/CdZnS QD over time. (d) PL lifetime of the single InAs/CdZnS QD in panel (c) during emission states of various intensity: high, intermediate, and low emission intensity conditions (colors of curves correspond to the shaded region in panel c, see the reference). Reprinted with permission from ref. 168 Copyright 2014, American Chemical Society.

Auger recombination (AR) is a three-particle non-radiative process, where the energy of an electron–hole pair is transferred to either an electron or a hole instead of emitting a photon.^155^ AR is frequently studied *via* TA or PL lifetime analyses.^156^ Since InAs QDs show very short Auger lifetimes (*τ*_AR_, <50 ps^148^), which can limit their optoelectronic applications, it is necessary to suppress AR to employ them in lasers,^157–161^ LEDs^162–164^ and single-photon sources.^165^ So far, in the literature there are reports on AR rates between 10 and 30 ps for InAs QDs, thus suggesting that AR is the main recombination channel of bi-excitons.^150,151^ Spencer *et al.* in 2019 performed pump-fluence-dependent absolute pump-probe transients of InAs QDs employing a beam scanning technique to limit the repetitive excitation.^151^ Through this advanced technique, they identified a *τ*_AR_ of 26 ± 5 ps for their 6.2 nm sized InAs QDs (Fig. 8c). Importantly, such value is consistent with what reported by Schaller *et al.* for 4.3 nm InAs QDs (*τ*_AR_ = 8.3 ps) considering QD volume scaling.^148,166^ Indeed, it is possible to slow down this process by improving the core/shell interfacial layer and by developing band engineering strategies. For that, Sagar *et al.* developed a continuously graded thick CdSe_*x*_S_1−*x*_ shell on InAs QDs to increase the degree of charge carrier delocalization from the core to the shell.^143^ This delocalization can reduce the electron–hole Coulombic interaction and slow down the Auger recombination to ∼105 ps, yielding a PLQY of 30% with emission at 1320 nm.^143^ On the other hand, Sagar *et al.* did not clearly demonstrate the influence of the graded shelling on the photophysical properties of InAs QDs.

### Single QD PL

Single InAs QD PL has been mainly addressed by the works of Correa *et al.* and Bischof *et al.*^167,168^ Here, the limited number of reports is due to the weak NIR emission of individual InAs QDs whose observation requires superconducting nanowire single-photon detectors. In 2012, Correa *et al.* performed PL measurements on single InAs/CdSe core/shell QDs where they observed two state blinking with an emission spectra centered at 1300 nm.^167^ Following that seminal work, the group reported further measurements on InAs/CdZnS^168^ in 2014, where they were able to perform a detailed analysis of the emission dynamics. Here, they observed PL blinking from 24 individual InAs/CdZnS QDs. Some of the QDs demonstrated binary blinking (switching between states of high intensity and low intensity, as observed in CdSe),^169^ while other QDs exhibited an intermediate intensity. The varying blinking behavior was accompanied by a radiative lifetime ranging from 50 to 200 ns and the authors could correlate a decrease of radiative lifetime to a decreased emission intensity (*i.e.* high, intermediate and low intensity states, Fig. 8c and d). Antibunching measurements performed on the very same QDs allowed the identification of the biexciton PLQY, which was found to vary from <1% up to 43%.^168^

## Applications

### Bioimaging

**Fig. 9:**
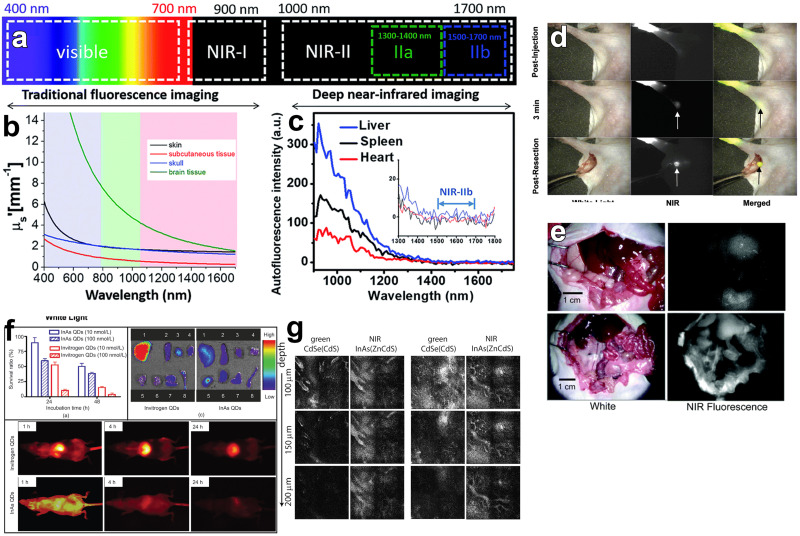
***In vivo* bioimaging using InAs based QDs emitting in NIR-I region**. (a) Spectral ranges of fluorescence imaging. (b) Reduced scattering coefficient (μs) of the various tissue types: skin (black), brain tissue (green), skull (blue) and subcutaneous tissue (red). (c) Auto-fluorescence spectrum of *ex vivo* mouse liver (blue), spleen (black) and heart (red) tissue. Reprinted with permission from ref. 176, Copyright 2018, The Royal Society of Chemistry. (d) Images of injected oligomeric phosphines capped InAs_0.82_P_0.18_/InP/ZnSe core/shell/shell QDs taken by white light, NIR fluorescence, and color/NIR merge, respectively. Reprinted with permission from ref. 141 Copyright 2005, American Chemical Society. (e) InAs/ZnSe core/shell QDs capped with DHLA (up) and DHLA-PEG (down) used for bioimaging. Reprinted with permission from ref. 73 Copyright 2006, American Chemical Society. (f) Cytotoxicity and bioimaging of mice major organs *via* 3-MPA capped InAs/InP/ZnSe core/shell/shell QDs. Reprinted with permission from ref. 74 Copyright 2008, Springer Nature. (g) Images of the InAs/ZnCdS and CdSe/CdS core/shell QDs injected intravenously and imaged simultaneously in a mammary tumor in a mouse (scale bar: 100 μm). Reprinted with permission from ref. 29 Copyright 2010, American Chemical Society.

Semiconductor QDs have been widely used for biomedical applications such as bioimaging,^170,171^ biosensing^172^ and neural photostimulation.^63,173–175^ InAs QDs are of interest for bioimaging and biomedical labeling since they can be both excited and detected in the NIR-II region of the spectrum (1000–1700 nm) (Fig. 9a), where tissue absorption and auto-fluorescence is minimal (Fig. 9b and c).^124,176,177^ Furthermore, InAs QDs are RoHS-compliant and less toxic than Cd-, Pb- and Hg-based materials, as already mentioned. In this regard, Kim *et al.* successfully exploited InAs QDs in a sentinel lymph node mapping experiment for the first time.^141^ In details, they synthesized InAs_*x*_P_1−*x*_/InP/ZnSe alloyed QDs (by using TMS-As and TMS-P reagents) and capped them with oligomeric phosphines. These QDs had a small hydrodynamic size (∼12 nm), aqueous stability and PLQY of 3.5% in water. Despite their low PLQY, 150 pmol of subdermally injected InAs_0.82_P_0.18_/InP/ZnSe alloyed QDs could be easily detected by bioimaging systems since they can enter the lymphatics and migrate within 1 minute to the sentinel node (Fig. 9d). In another study, the same group employed dihydrolipoic acid conjugated to a short poly(ethylene glycol) (DHLA-PEG) as surfactants for more emissive InAs/ZnSe core/shell QDs (PLQY of 6–9% in water).^73^ In contrast to oligomeric phosphines, QDs capped with DHLA-PEG can be used as efficient and specific *in vivo* labels since they can circulate for a longer time (up to 5 minutes) and migrate out of the blood vessels and into the interstitial fluid (Fig. 9e). Another possible capping agent for InAs QDs is 3-mercaptopropionic acid (3-MPA), which was used by Xie *et al.* for *ex vivo* fluorescence imaging of major organs of mice (Fig. 9f).^74^ In terms of imaging depth, Allen *et al.* could clearly image tumor vasculature (E0771 mammary) even up to 200 μm using InAs/ZnCdS core/shell QDs (Fig. 9g).^29^

**Fig. 10:**
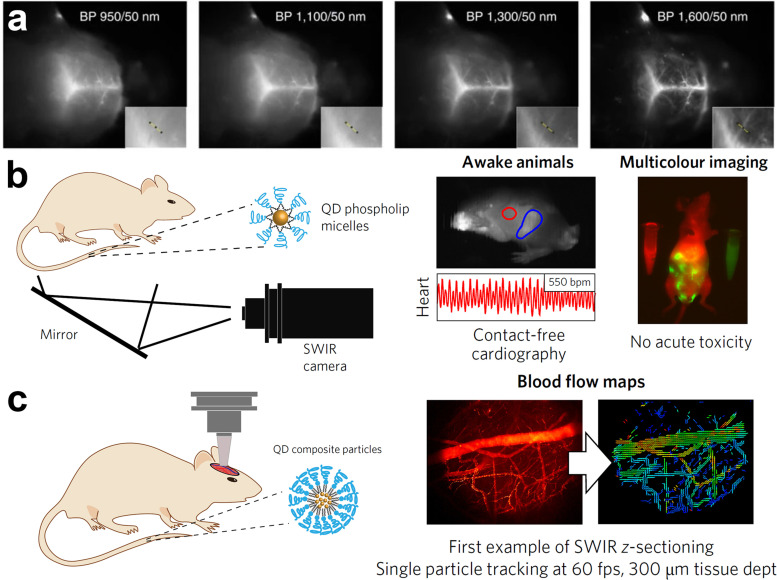
***In vivo* bioimaging using InAs based QDs emitting in NIR-II region**. (a) Fluorescence imaging of mouse brain vasculature through intact skin and skull using a mixture of InAs/CdSe/CdS core/shell/shell QDs emitting at 970 nm, 1100 nm, 1300 nm and 1600 nm. Reprinted with permission from ref. 30, Copyright 2016, Springer Nature. (b) Bioimaging in the ventral orientation at 30 fps with InAs QDs functionalized with phospholipid micelles under excitation of 808 nm allowed imaging of vital signs such as heart rate (*λ*_Exc_ = 808 nm). (c) High-resolution and high-speed SWIR intravital imaging using InAs composite QDs in a mouse with a cranial window to generate flow maps of microvascular networks (*λ*_Exc_ = 808 nm; longpass: 1000 nm). Reprinted with permission from ref. 77, Copyright 2017, Springer Nature.

In contrast to previous QDs emitting below 900 nm, Franke *et al.* synthesized InAs/CdSe/CdS core/shell/shell QDs emitting at 970 nm, 1100 nm and 1300 nm.^30^ They injected the mixture of these QDs into an anaesthetized mouse *via* the tail vein and the vasculature of the mouse brain was imaged through the intact skin and skull using diffuse 808 nm excitation. Their results showed that longer imaging wavelengths enhance the spatial resolution of bio structures by increasing the signal to background ratio (Fig. 10a). Furthermore, they saw a tradeoff between increased resolution and decreased imaging speed at longer imaging wavelengths. These points indicate the need for NIR-II emitting InAs QDs with narrow emission and high PLQYs at the red edge of short-wavelength infrared region (SWIR) detectors. Similarly, Bruns *et al.* used InAs/CdSe/CdS (*λ*_em_ = 1080 nm) and InAs/CdSe/ZnSe (*λ*_em_ = 1280 nm) core/shell/shell QDs and functionalized them with three distinct surface coatings (phospholipid micelles, lipoproteins and composite) to tailor the physiological properties for specific SWIR imaging applications.^77^ Surface functionalization with phospholipid micelles allowed long blood circulations times and thus enabled assessment and quantification of heart rate and respiration of both sedated and awake mice (Fig. 10b). By tracking individual composite InAs particles during intravital microscopy, they could directly identify arteries/veins, and quantify blood flow in the vasculature of the brain (Fig. 10c).

### Electronics

It is possible to fabricate stable p–n homojunction-based devices such as field-effect transistors (FETs) by controlling the carrier type and transport in QD solids.^178,179^ Narrow band-gap III–V semiconductors (*e.g.* InAs, InSb and InN) are of interest for FETs due to their high electron mobility (2 × 10^4^ cm^2^ V^−1^ s^−1^ at 300 K for InAs^16^), high chemical stability and, most importantly, RoHS-compliance. Furthermore, surface dangling bonds of these semiconductors pin the InAs surface Fermi level in the conduction band and create an electron accumulation layer on the surface.^16,38,180^ Likewise, InAs QDs show n-type behavior which has been attributed to electron donating surface states, as probed by Steiner *et al.* using scanning tunneling spectroscopy.^145^

**Fig. 11:**
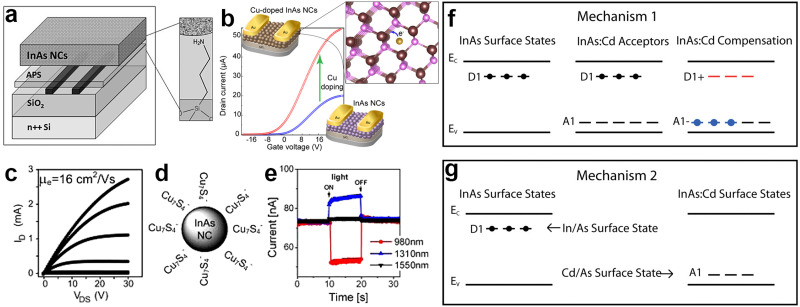
**InAs QD based field effect transistors (FETs)**. (a) Schematic of InAs QD based FET consisting of heavily doped silicon gate, 100 nm dry thermal silicon oxide, Cr/Au (5 nm/50 nm) source/drain contacts, 3-(trimethoxysilyl)propylamine (APS) self-assembled molecule and InAs QD film as the semiconductor. Reprinted with permission from ref. 181, Copyright 2010, John Wiley and Sons (b) illustration of the InAs and Cu:InAs based FET and enhancement of the mobility upon Cu doping. Reprinted with permission from ref. 129 Copyright 2019, American Chemical Society. (c) Plots of the drain current (ID) *vs.* drain-source voltage (VDS) measured at different gate voltages (VG), (d) schematic representaion and (e) ambipolar photoresponse of FET device based on Cu_7_S_4_ capped InAs QDs. Reprinted with permission from ref. 182 Copyright 2013, American Chemical Society. (f and g) Two approaches for the p-type doping of Cd:InAs QDs. Reprinted with permission from ref. 179 Copyright 2010, American Chemical Society.

In 2010, Soreni-Harari *et al.* fabricated FETs using colloidal InAs QDs for the first time and investigated the effect of the interface region within the active layer of InAs through a dedicated surface and film treatment (Fig. 11a).^181^ Firstly, they replaced the native TOP molecules capping InAs QDs with aniline *via* a ligand exchange procedure, then assembled QDs onto the amine linking sites of 3-(trimethoxysilyl)propylamine (APS)-functionalized surface, and finally crosslinked them with ethylenediamine (EDA). Using this approach, they achieved high quality films exhibiting an *I*_on/off_ of 10^5^ and a linear regime mobility of 1.2 × 10^−5^ cm^2^ V^−1^ s^−1^. Their InAs QDs based device showed n-type conduction characteristic and no p-channel conductance. Recently, Tripathi *et al.* showed that copper doping of InAs QDs improves the n-type mobility by 150% due to the surface trap passivation (Fig. 11b).^129^ In another study, Liu *et al.* reported a performance enhancement of InAs based FETs using InAs QDs capped with molecular metal chalcogenide complexes (MCCs).^182^ The electron mobility of InAs QDs was enhanced up to a factor of 10^6^ when replacing the native organic ligands with inorganic anions such as Cu_7_S_4_^−^ (Fig. 11d and f). The FETs were fabricated by depositing ∼27 nm of Cu_7_S_4_^−^ capped InAs QDs on silicon wafer obtaining an electron mobility of 16 cm^2^ V^−1^ s^−1^ in the linear regime and 14.8 cm^2^ V^−1^ s^−1^ in the saturation regime (*a* value which is orders of magnitude higher than previously reported for InAs-^181^ and CdSe-^183–186^ FETs). The electron mobility increased with increasing annealing temperature and rapid thermal annealing (RTA) gave the highest mobility (Fig. 11c).

In order to manipulate the charge carrier type of InAs QDs and n-type to p-type conversion for FETs, two approaches were reported. In the first approach, Cd atoms were incorporated at indium sites in the lattice, leading to acceptor states above the valence band maximum and inducing a p-type semiconductor behavior (Fig. 11f).^179^ In the second approach, Cd atoms were doped on the InAs surface, acting as substitutional p-dopants (see “synthesis of InAs-based alloyed/doped QDs” subsection for more details) (Fig. 11g).^36,179^ Furthermore, Cd doping created a protective layer and increased the chemical stability against oxidation under ambient atmosphere. The characteristics and performance of the InAs based FETs are summarized in Table 3.

**Table 3:** FET characteristics of the reported InAs based FET devices. All of them are n-type except Cd:InAs, which is p-type

QD type	Ligand	Layer thickness (nm)	*μ*_lin_ (cm^2^V^−1^ s^−1^)	*μ*_sat_ (cm^2^V^−1^ s^−1^)	*I*_on/off_	Ref.
InAs	Cu_7_S_4_^−^	∼27	16.0	14.8	∼10^3^	182
InAs	DOA	50	1.2 × 10^−4^	1.3 × 10^−4^	10^3^	27
InAs	Br	50	1.4 × 10^−3^	1.7 × 10^−3^	10^4^	27
InAs	MPA	50	2.0 × 10^−3^	2.5 × 10^−3^	10^4^	27
InAs	TOP	n/a	2.5 × 10^−6^–5 × 10^−6^	n/a	10^3^	181
InAs	EDA	n/a	8.5 × 10^−6^–1.2 × 10^−5^	n/a	10^5^	181
InAs	EDT	35	0.08	0.07	10^5^	129
Cu:InAs	EDT	35	0.18	0.15	10^4^	129
Cd:InAs	EDT	35	1.5 × 10^−3^	n/a	n/a	36

### Optoelectronics

**Fig. 12:**
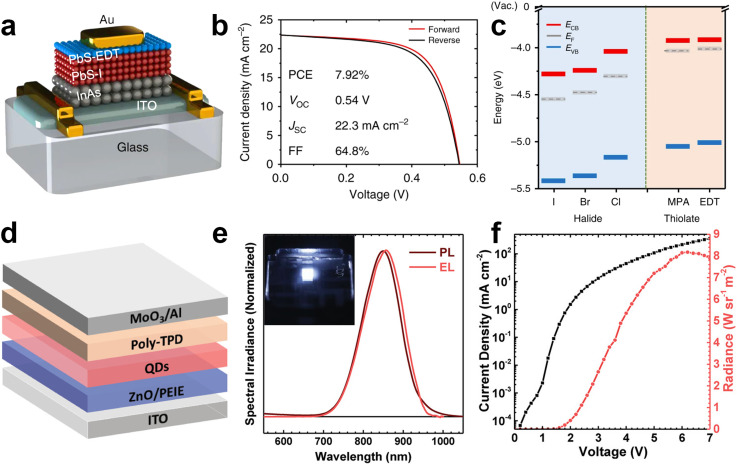
**InAs QDs for photovoltaics and NIR LEDs**. (a) Schematic and (b) *J*–*V* of the n-type InAs based p–n junction photovoltaics. (c) Energy levels *E*_CB_, *E*_VB_, and *E*_F_ (*vs.* the vacuum level) of InAs QD films capped with different ligands. Reprinted with permission from ref. 27, Copyright 2018, Springer Nature. (d) Schematic of ITO/ZnO/PEIE/In(Zn)As@In(Zn)P@GaP@ZnS QDs/Poly-TPD/MoO_3_/Al NIR LED and (e) EL and PL spectra of In(Zn)As/In(Zn)P/GaP/ZnS QDs. (Inset: NIR image of the operating NIR LED). (f) Current density and radiance *versus* voltage characteristics of InAs based NIR LED. Reprinted with permission from ref. 95, Copyright 2020, John Wiley and Sons.

InAs QDs have only marginally being tested in common optoelectronic devices such as LEDs^95,144,187,188^ and photovoltaic cells.^27^ Yet, to date, there have been no reports on amplified spontaneous emission or lasing from InAs QDs. Regarding photovoltaic applications, Song *et al.* obtained power conversion efficiency of 7.92% from a p–n junction between n-type InAs and p-type PbS colloidal QD layers (Fig. 12a and b).^27^ They assembled defect-controlled conductive InAs films *via* a two-step surface modification. They stripped the native ligands (oleic acid) from InAs QDs with NOBF_4_ and replaced them with either 1,2-ethanedithiol (EDT), 3-MPA, I, Br or Cl. Interestingly, the presence of these new ligands led to a shift of the band edges of the QDs up to 0.4 eV. The dipole moment of the ligand/QD interface and the intrinsic dipole moment of the ligand were employed to modify the energy levels.^189^ Ligand-induced surface dipoles can indeed control the energy levels in addition to quantum confinement-controlled bandgap modification. Halide ligands create deeper band energy levels since they have large interface dipole moments as reported for PbS QDs.^190^ Among them, InAs capped with I^−^ showed the deepest energy level (−5.4 eV). On the other hand, EDT capped InAs exhibited *E*_VB_ around −5.1 eV possibly due to the offset direction of the interface dipole moment and the ligand intrinsic dipole moment (Fig. 12c). These findings reveal that rational ligand engineering holds promise for the fabrication of defect-controlled conductive InAs based assemblies and efficient photovoltaics.

Electroluminescence (EL) has been one of the prime applications of colloidal QDs. While QD LEDs emitting in the visible spectral region are well-established,^2,164,191–193^ NIR QD LED are still not fully developed. InAs QDs represent one of the most appealing QDs for the fabrication of NIR LEDs, since they are fully RoHS-complaint and capable of fully covering the NIR spectrum. Due to the low in-film PLQY and high nonradiative processes, there are only few reports on the application of InAs in EL devices. In 2002 Tessler *et al.* reported the very first NIR LED based on InAs QDs where they incorporated InAs/ZnSe core/shell QDs in a conjugated polymer matrix exhibiting energy transfer to the NIR emitting QDs. The hybrid organic/inorganic NIR LED showed external quantum efficiency (EQE) of 0.5% and emission around 1200 nm.^144^ A second example is the recent work from Wijaya *et al.*, where they built an inverted NIR LED based on In(Zn)As/In(Zn)P/GaP/ZnS core/shell/shell/shell, ZnO electron transport layer and a poly(*N*,*N*′-bis-4-butylphenyl-*N*,*N*′-bisphenyl)benzidine (Poly-TPD) hole transport layer (Fig. 12d).^95^ They demonstrated a NIR LED emitting in the 800–900 nm range with an EQE of 4.6% and radiance of 8.2 W sr^−1^ m^−2^ (Fig. 12e and f). By employing similar core/shell/shell/shell QDs, but featuring a larger InAs core, the same group reported NIR LEDs with an EQE up to 13.3% at 1006 nm.^187^ It should be noted here that such performance was achieved using QDs with a relatively complex architecture, which requires an elaborate synthesis protocol based on both TMS-As and TMS-P. On the contrary, De Franco *et al.* recently demonstrated a stable NIR LED based on much simpler InAs/ZnSe QDs synthesized *via* amino-As and reported EQE of 5.5% at 947 nm.^188^ These works highlight the potential of InAs QDs for NIR LEDs; yet, they point out as well the necessity to develop efficient InAs QDs emitting in NIR-II region and device engineering for increasing the EQE of NIR LEDs.

## Outlook & future aspects

Herein, we have reviewed comprehensively the progress made so far on the colloidal synthesis and applications of InAs QDs. These results enrich the field of NIR QDs and suggest a promising future for InAs QDs since it is the only RoHS-compliant available semiconductor that can absorb and emit in the whole range of NIR. However, the overall toxicity of InAs QDs must be assessed in the future. For instance, it is still unknown whether the cytotoxicity of InAs QDs originates from the released In^3+^ and As^3−^ ions upon etching or from the intracellular distribution of QDs and the associated nanoscale effects as reported for Cd-based QDs.^194^ From the synthesis point of view, the research on colloidal InAs QDs is a hot topic as improvements in the synthesis are required to achieve further control over the size, size distribution and optical properties enabling a variety of optoelectronic/electronic applications. These issues need to be addressed before InAs QDs can gain equivalent attention in NIR technology as lead chalcogenides. In particular, the low PLQY of InAs QDs currently limits their application in NIR LEDs and so far, most of the studies on NIR LED were focused on lead-containing QDs^7^ or perovskite host matrix showing EQE up to 16%.^195^ In terms of optical properties, it is necessary to elucidate further the carrier dynamics and assess the exciton fine structure as well as the origin of the dark exciton radiative recombination in core and core/shell InAs QDs; along the same line of what reported for InP QDs (the “neighboring” III–V semiconductor system, see ref. 196). Furthermore, we also have little knowledge about the surface chemistry of InAs QDs and how ligands can affect their surface energy, nucleation, growth, and shape evolution. So, comprehensive XPS, FTIR, and NMR studies are needed since surface chemistry engineering is crucial to create “strain-free” core/shell systems (to increase their PLQY) and facilitate the development of InAs based NIR LEDs.^100,188^ Future progress on novel precursors and synthetic methodologies should not only pursue the goal of making more luminescent InAs QDs, but also reduce non-radiative processes such as Auger recombination, specifically for QD-based optical gain media in the NIR range. In addition, the photo- as well as chemical-stability of InAs must be investigated in detail. On the other hand, InAs QDs can be attractive for luminescent solar concentrators (LSCs) due to their tunable absorption over the entire visible range as well as the closely-matched PL with the low-energy part of the EQE spectrum of the silicon-based photovoltaic cell. However, efficient LSCs demand reduced reabsorption while maintaining a high PLQY, so effective material design and device engineering are still required for InAs QDs as performed previously for InP-^2,197–199^ and CISeS-^200^ QD based LSCs. Taken together, the urgent need for RoHS-compliant IR semiconductors creates a powerful motivation for the QD community to work on efficient InAs QDs. On the other hand, the range of 1550–1600 nm is important for telecommunication as well as quantum secure communication^28^ and deep tissue imaging^34^ and so far only lead- (PbSe^201,202^ and PbTe^203^) and mercury-based (HgTe^204^ and HgSe^205^) QDs can emit efficiently in this range. Shifting the emission wavelength to the 1550–1600 nm range is therefore another challenge for future studies, since the longest reported emission wavelength of colloidal InAs QDs is around 1500 nm.^99^ The synthesis of alloyed In–As–Sb QDs could be one approach to tune the PL emission range to longer wavelength. Large-scale production is yet another issue for InAs QDs considering the hazardous and expensive arsenide precursors as well as limited supply of indium element. For that, commercially available and non-hazardous precursors must be developed; while non-injection organometallic synthesis approaches to prepare large quantities require extensive investigation. Last but not least, the identification of structural defects of InAs synthesized *via* either TMS-As or amino-As is still a challenge, so strategies to cure such defects have to be explored as reported for GaAs QDs.^206^ Finally, progress on InAs QDs relies on novel synthesis techniques as well as effective post-synthesis modifications, which provide the keys to efficient optical and electronic performance.

## Abbreviations

AR: Auger recombination
amino-As: Tris(dimethylamino) arsine
APS: 3-(Trimethoxysilyl)propylamine
AsTMGe_3_: Tris(trimethylgermyl)arsine
AsH_3_: Arsine
AsPh_3_: Arsenic silylamide
CM: Carrier multiplication
Cd: Cadmium
CdS: Cadmium sulfide
CdSe: Cadmium selenide
C_6_H_15_In: Triethylindium
DIBAL-H: Diisobutylaluminum hydride
DMAH: Dimethylaluminum hydride
DMEA-AlH_3_: Dimethylethylamine complex
DOA: Dioctylamine
EDT: 1,2-Ethanedithiol
EL: Electroluminescence
EQE: External quantum efficiency
eV: Electron volt
FET: Field effect transistor
HF: Hydrofluoric acid
In(Ac)_3_: Indium acetate
InAs: Indium arsenide
InCl_3_: Indium chloride
InP: Indium phosphide
(iPrDMSi)_3_As: Tris(isopropyldimethylsilyl) arsine
LA: Lauric acid
LED: Light emitting diode
LiEt_3_BH: Lithium triethylborohydride
LSC: Luminescent solar concentrators
MA: Myristic acid
MBE: Molecular beam epitaxy
MCC: Metal chalcogenide complexes
[(Me_3_Si)_2_N]_2_AsCl: Triphenylarsine
ML: Monolayer
MPA: 3-Mercaptopropionic acid
NC: Nanocrystal
NIR: Near infrared
NW: Nanowire
NR: Nanorod
OAm: Oleylamine
ODE: Octadecene
PA: Palmitic acid
PbS: Lead sulfide
PL: Photoluminescence
PLQY: Photoluminescence quantum yield
PNC: Prenucleation cluster
P(NEt_2_)_3_: Tris(diethylamino)phosphine
QD: Quantum dot
RES: Reticuloendothelial system
RoHS: Restriction of hazardous substances
SWIR: Shortwave infrared
TA: Transient absorption
(TEGe)_3_As: Tris(triethylgermanyl)arsine
TOP: Trioctylphosphine
TMGe_3_As: Tris(trimethylgermyl)arsine
TMS-As: Tris(trimethylsilyl)arsine
RTA: Rapid thermal annealing
ZnSe: Zinc selenide

## Conflicts of interest

There are no conflicts of interest to declare.

## Supplementary Material
